# Transfemoral Socket Fabrication Method Using Direct Casting: Outcomes Regarding Patient Satisfaction with Device and Services

**DOI:** 10.33137/cpoj.v3i2.34672

**Published:** 2020-11-23

**Authors:** W.R. Marable, C Smith, B.Þ. Sigurjónsson, I.F. Atlason, G.A. Johannesson

**Affiliations:** 1 Össur Americas, Foothill Ranch, California, USA.; 2 Össur HF, Reykjavik, Iceland.; 3 Quick Lookup, Reykjavik, Iceland.; 4 TeamOlmed, Stockholm, Sweden.

**Keywords:** Transfemoral amputation, Amputee, Prosthesis, Socket, Interface, Outcome measure, Satisfaction, Direct casting

## Abstract

**BACKGROUND:**

Direct Socket for transfemoral (DS-TF) prosthetic user is a novel method of fabricating a laminated interface on to the residual limb but requires different training, production method and service model than what most prosthetists are familiar with. This method and model may improve patient satisfaction by enabling interface fabrication and delivery in one visit.

**OBJECTIVES:**

Document patient satisfaction regarding DS-TF interface versus the prosthetic users’ previous socket in terms of interface function and the clinic service model.

**METHODOLOGY:**

In this longitudinal study (from July 2018 to April 2020), the DS-TF was implemented in six prosthetic clinics across the United States. Certified prosthetists (CP) and assistants were trained using a standard protocol. 47 prosthetic users participated, both those in need of a new socket and those without need. Two modules from the Orthotics and Prosthetics Users’ Survey (OPUS), involving questions related to satisfaction with the Device and Services, was used to evaluate each DS-TF user outcome vs. baseline. The only part of the prosthesis that was replaced was the interface, except in 2 cases.

**FINDINGS:**

Each DS-TF interface was fabricated, fit and delivered in a single clinic visit. At 6-months follow-up, 38 users reported an average of 29.8% increase in satisfaction with their new interface compared with original, and a 14.8% increase in satisfaction with the services they received from the clinic in providing of the new prosthesis vs. their original prosthesis. The main outcome increases were between baseline (initial fitting) and 6-week follow-up and remained consistent after 6 months. This improvement was consistent irrespective if the user needed a new socket for clinical reasons or not.

**CONCLUSIONS:**

This study shows that after a standardized training and implementation, the DS-TF fabrication process including a new interface, improves the user’s satisfaction with their prosthetic device and services.

## INTRODUCTION

Transfemoral (TF) amputation(s) is a devastating procedure for any person and expensive in terms of acute healthcare cost^1^ and rehabilitation cost.^2,3^ TF amputation highly restricts the amputee’s overall mobility^4^ and requires a good functional prosthesis, especially the interface (i.e. the socket, the liner and its suspension) to enable the amputee to regain as much of their previous mobility as possible.^5,6^ TF amputation is also associated with longer amputee rehabilitation time compared with transtibial (TT) amputation and more need of assistance, especially in elderly patients.^7^ TF amputees report their general health related quality of life to be lower than that of non-amputees or TT amputees. Furthermore, specific problems have been related to the use of TF prostheses, including limited hip joint range of motion that restricts users comfort or ability to perform daily activities such as sitting, walking, picking objects up from the floor, and tying their shoes.^8,9^ Despite new materials and technology, the most frequently reported problem with the usage of TF prosthesis is still sores/skin irritation from the interface.^10,11^

Prosthetic fitting of TF amputees has always been a challenging and time-consuming procedure that requires the user (and often family member or care giver) to make on average 4 visits to a prosthetic clinic.^12^ Between 6 and 16 weeks pass from amputation to delivery of a TT or TF prostheses in high-income countries.^13–15^ Fabricating a definitive TF interface has been reported to take at least 14 days using a traditional laminated interface process of casting, check socket(s) and laminated definitive interface.^16^ Reducing the number of days in rehabilitation can reduce amputee rehabilitation costs by up to 25%.^2^ It has also been demonstrated that in patients amputated due to vascular reasons, functional mobility level declines as the number of days between amputation and start of rehabilitation increases. In the same study shorter time to prosthetic fitting was demonstrated to lead to improved functional outcomes six months after amputation.^17^

Although many interesting new socket designs have been introduced to the market in the last 50 years, few studies have documented long term outcomes and/or quantitative data of a specific socket design.^11^ Most published studies focus on TF amputee gait, socket design, suspension and/or function of the components including less than 15 amputees.^8,18–20^ Kahle et al found that different socket designs may have an effect on gait speed and risk of falling.^8^ Fatone et al., have described a new interface and the fabrication methods used to produce a TF socket, including outcomes from two cases.^21,22^ Kahle et al., investigated the trimline level and compared outcomes between socket designs in 15 cases, although only in a clinical setting.^18^

The socket designs in these studies are mainly grounded on the prosthetist’s experience, with few measurable design parameters or description of how the design is intended to affect and interact with the user’s residual limb during the gait cycle.^11^ Various terms have been proposed to describe the way forces are transferred between the residuum and the socket, the biomechanical principles upon which these terms are based are ill-defined.^23^ Most recognized are terms used to describe the axial and transversal stabilization, e.g. Ischial Containment or Inter Ramus Containment sockets.^11^ Contact between this part of the hip bone (ischium and ramus inferior) and the socket can only be obtained during stance phase, which is approximately 0.6 sec of a full 1.07 sec gait cycle.^24^ During the rest of the gait cycle the ramus is moving in and out of the socket with little or no socket interaction. The position and effect of the “ischial-ramus containment” has not been revealed in studies except in theory.^25^ When this support or function is presumed to be lacking the term ”sub-ischial socket” has been used.^26^

**LIST OF ABBREVIATIONS**
- **CP:** Certified Prosthetist
- **CSD:** Client Satisfaction with Device
- **CSS:** Client Satisfaction with Services
- **DS-TF:** Direct Socket for transfemoral
- **DS-TT:** Direct Socket for transtibial
- **ISO:** International Organization for Standardization
- **ISO/TC:** 168 Prosthetic and Orthotics working group within ISO
- **ISPO:** International Society for Prosthetics and Orthotics
- **OPUS:** Orthotics and Prosthetics User’s Survey
- **O&P:** Orthotics and Prosthetic
- **TF:** transfemoral
- **TT:** transtibial
- **6WFU:** six-weeks follow-up
- **6MFU:** six-month follow-up

The problem with this “definition” is that a transtibial or toe prosthesis could also be referred to as sub-ischial socket. This lack of detail and consistency in definitions prevents objective comparison.^27^ In an effort to provide detail and resolve the inconsistency, ISO standard 13405-2:2015 contains recommendations on how to systematically describe an interface and provides a base for comparison between different interface design and function.^23^

A method of fabricating a finished laminated TT interface directly on the patient’s residual limb has been on the market since 1996. First introduced as ICEX by Össur HF of Iceland, ICEX was based on the inventor’s philosophy of pressure casting^28^ and was included in a variety of studies related to user satisfaction, cost and function.^29–32^ Since then ICEX was improved to Modular Socket System (MSS) in 2005,^31^ and finally to Direct Socket in 2018.^33^ This system enables a prosthetist to fabricate a custom-made interface directly on the TT residual limb in a single visit. The lesson learned from the use of DS-TT in Scandinavia for over two decades and the finding related to the process, e.g. shorter rehabilitation time, demanded a solution for TF level.^13^

A TF version of Direct Socket (DS-TF) began testing in 2016 in select Scandinavian clinics.^30,31^ The proximal portion of a DS-TF interface design differs significantly from the proximal portion of sockets typically called Ischial Containment or Inter Ramus Containment sockets, as the proximal part of the DS-TF includes a size-specific silicone brim. The method of fabricating directly on the residual limb requires a different approach to prosthetist training and fabrication compared to the socket fabrication process most prosthetists apply today.^33^

The primary aim of this study was to collect data on prosthetic users satisfaction regarding DS-TF interface in terms of both interface function and the clinic service model (i.e. one patient visit to the clinic for fabrication of custom TF interface, prosthesis assembly, alignment, gait analysis, and delivery).

## METHODOLOGY

**Figure 1:**
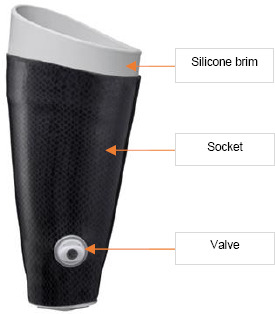
Direct Socket

**Figure 2:**
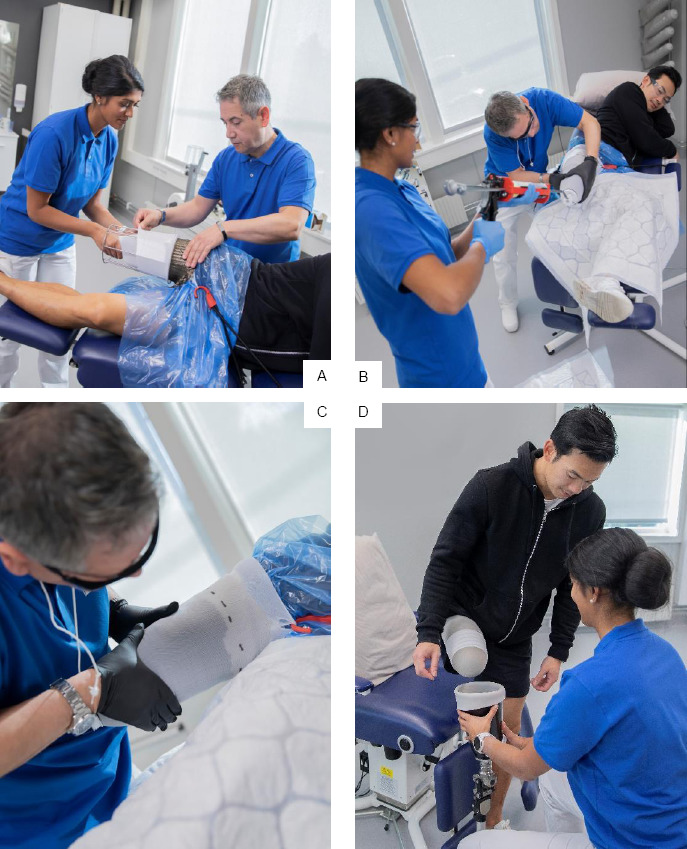
DS-TF process A) application of material, B) resin injection, C) moulding of resin, D) first fit and alignment check.

In this study, a new direct casting procedure for TF amputees called Direct Socket TF (DS-TF),^34^ was implemented in six different prosthetic clinics across the United States. The product includes all materials and tools to make a definitive socket. In the DS-TF fabrication process, a special 2.5mm casting liner is rolled onto the residual limb followed by a protective silicone sheath over the casting liner to prevent the resin from contacting the residual limb. Next, a size-specific silicone brim is placed at the proximal part of the limb. A glass or basalt fabric with pre-attached distal adapter is then rolled on the length of the limb. A second protective sheath is applied on the outside of the fabric and a two-part resin is injected through the distal adapter (Figure 1, Figure 2) to approximately ½ to ¾ of the fabric length. The resin is absorbed through the fiber and kept isolated from the amputee by the two silicone sheaths on each side of the fabric. Over the next 10-15 minutes the resin undergoes an exothermic reaction as it hardens, during which the CP can mold and shape the socket wall around the residual limb muscles that are relaxed or contracted as directed by the CP. The amputee can feel the warming socket, but socket temperature does not exceed 37.5 degree Celsius, a typical adult body temperature.

### RESULTS

According to ISO 13405-2:2015 section 5, (5.1.2.1 General) the force-transmission properties of DS-TF can be described, as follows:

- (5.1.2.2) AXIAL STABILIZATION: the direct socket shape conforms to the shape of the femur, and soft tissues about the femur, causing even compression of the soft tissue, which creates axial stabilization (see also 5.1.3).
- (5.1.2.3) TRANSVERSE STABILIZATION: even tissue compression created during the direct lamination process increases soft tissue density, creating anteroposterior, mediolateral, and rotational stabiliz-ation (see also 5.1.3).
- (5.1.2.4) SUSPENSION: to minimize the axial movement, several suspension methods may be used; preferably a Seal-In liner which creates partial distal vacuum, a locking liner with lanyard or pin can also be used.
- (5.1.3) STIFFNESS: The socket is laminated with a distal 4-hole adapter and a proximal brim made of flexible silicone. This makes the socket flexible proximally while most of the socket is rigid. The flexible circumference silicone brim encompasses and compresses the proximal thigh muscles when contracted, thereby stabilizing the hip at initial contact, loading response, mid-stance, and terminal-stance, and creating axial and transverse stabilization. During the rest of the gait (pre-, initial-, mid-, and late swing) the brim is only following the hip movement.

The selection criteria for study principle investigators included a Certified Prosthetist (CP) with more than 5-years clinical experience in serving TF amputees with interest in improving patient outcomes and satisfaction, willingness to follow a defined novel fabrication protocol, and commitment to document outcome measures at defined time intervals. The inclusion criteria (*rationale*) is listed in Table 1.

**Table 1:** Amputee inclusion criteria (*rationale*)

Amputee inclusion criteria *(rationale)*	
•	50Kg< body weight < 160Kg *(the ISO validated weight limit of the DS-TF)*
•	Cognitive ability to understand all instructions and questionnaires in the study
•	Patients who have undergone a TF amputation > 1-year post amputation *(this was to avoid postoperative problems and/or adjustments related the initial prosthetic fitting of a new amputee)*
•	Older than 18 years
•	Willing and able to participate in the study and follow the protocol
•	Circular dimension of 40-65 cm at the crotch *(limited to available silicone brim sizes)*
•	Residual limb length at least 20 cm from ischium to distal end *(fabrication limitation of the DS-TF)*
•	Currently using a prosthetic liner *(this was to avoid potential confounding influence from transitioning an amputee from a skin fitting interface (i.e. without a liner), to an interface with a liner)*
•	Willing to use a silicone prosthetic liner as called for in Direct Socket Instructions For Use.^34^

### Training protocol

Direct Socket fabrication was performed by a two-person team consisting of a lead and an assistant (Figure 2). The lead was typically a CP while the assistant was commonly a prosthetic tech or certified prosthetic assistant. CP’s and assistants were trained in each CP’s clinic by certified Clinical Specialists using a standard protocol. Prior to starting training and fabrication, demographic/clinical information and measurements were documented to ensure subjects were within the clinical limitations of the study. Also, all necessary materials (sized for the scheduled amputees) were on hand, including Direct Socket Toolkit, Direct Socket Material Kits, Direct Socket casting liners and Direct Socket Fabrication Manual. All training and fabrication were completed following the step-by-step process of the Direct Socket Fabrication Manual.^34^ Amputees selected for clinician training were relatively uncomplicated without invaginations, unusual limb shapes or extreme alignments. After training, the Clinical training Specialist remained available to remotely consult with trainee CP’s as needed.

### Follow Up and Data Collection

**Figure 3:**
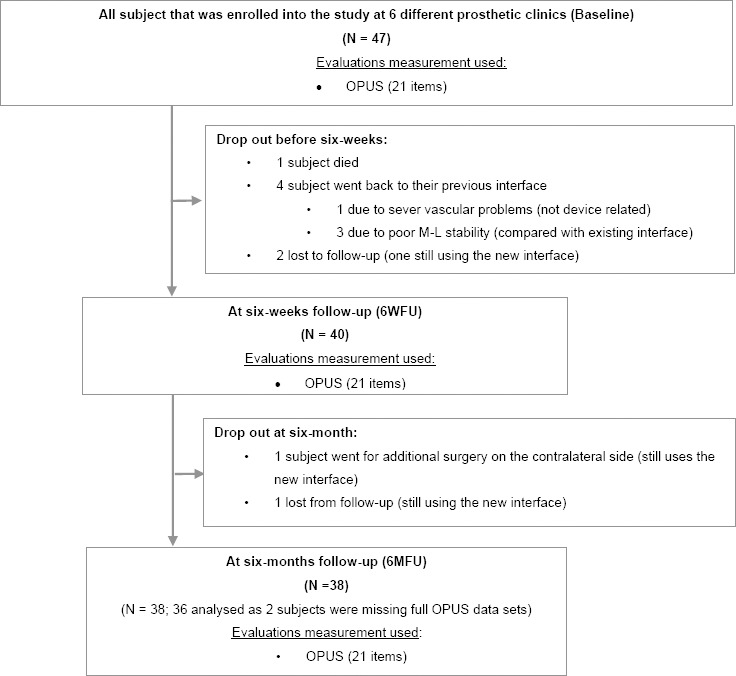
Flow chart of the investigation

Including the baseline, three time periods were used to assess outcomes (Figure 3). After 6 weeks subjects returned to the clinic and completed the same standardized surveys regarding their new prosthesis (6WFU). Subjects returned to the clinic again and completed the same surveys after 6 months as well (6MFU).

Prior to the fitting each amputee subject was asked to fill out a standardized survey regarding their existing prosthesis and the experience of the service that they previously received. After baseline the CP and assistant fabricated and delivered a new DS-TF interface and liners.

For the evaluations the Orthotics and Prosthetics User’s Survey (OPUS) was used. The OPUS is a set of self-reported outcome measures to be used within O&P clinics for the assessment of functional status, quality of life, and client satisfaction.^35^ The OPUS was originally developed in English and has been translated into multiple languages, including Spanish, Swedish and Slovenian. The OPUS has displayed good internal consistency and has been validated in US and Swedish populations.^36^ The OPUS instrument consists of five independent modules, two of which were used in this study: Client Satisfaction with Device (CSD) and Client Satisfaction with Services (CSS). The CSD and CSS include a total of 21 questions, scored on a 1 - 6, discrete scale:

*Strongly Agree, Agree, Neither agree nor disagree, Disagree, Strongly disagree and Don't Know/Not Applicable.*

The OPUS can be used on prosthesis and/or orthosis users. However, since this study was exclusively on prosthesis users, to prevent confusion the words “orthosis” and “orthotist” used in the original survey text were not included in this study’s user surveys.

The CSD Score is the sum of scores for items 1-11 (11 – 55 points) and relates to the function of the device and the user’s cost to acquire the device. Meanwhile the CSS Score is the sum of the scores for items 12-21 (10 – 50 points) and relates to the service the amputee received. A higher score indicates a better outcome. These raw scores were then converted to Rasch Measure (0 – 100 scale) and measure of variability is reported with the mean.^36^

The “Device” that is referred to in the questionnaire includes the complete prosthesis, of which questions 1, 3, 4, 8 and 9 only pertain to the interface. The DS-TF was delivered without any additional finishing components at baseline.

This study uses the K-scale system established in 1995, also called Medicare Functional Classification Levels (MFCL). The K levels divide lower limb amputees into five categories ranging from K level 0 (least mobile) to K level 4 (most mobile) intended to indicate a person’s rehabilitation potential.^37^

### Sample size calculation and statistical methods

A pretrial power analysis for the estimated required sample size was conducted using GPower^38^ version 3.1.9.6 and effect size was estimated based on published articles^9,39,40^ for the primary endpoint assuming a normally distributed amputee population. It was therefore expected that 38 subjects were required to complete the protocol with a power of 0,95 and α at 0,05. Drop-out rate was estimated at proximally 20% and therefore 47 subjects were recruited.

We used R version 4.03 (R-Studio Version 1.2.5033) and lme4^41^ to perform a linear mixed effects analysis of the relationship between the OPUS outcomes and clinical need. As fixed effects, we entered age, gender, and evaluation point (tested for interaction with “clinical need”) into the model. As random effects, we had intercepts for subjects and investigators, as well as by-subject and by-item random slopes for the effect of clinical need. P-values were obtained by likelihood ratio tests of the full model with the effect in question against the model without the effect in question. For comparison of individual OPUS items, the Benjamini & Hochberg method was used to control for Type I error due to multiple comparisons.^42^

## RESULTS

Between July 2018 and October 2019, 47 TF prosthetic users that fulfilled the eligibility criteria and agreed to participate in the study were enrolled. Ethical approval was obtained from Advarra^®^ IRB (CR00128417) and the investigation was registered at Clinical trials.gov NCT04312724. Signed informed consent was obtained from all participants.

Study subjects, included for data collection and analysis, consisted of prosthetic users fitted during the training of each CP on DS-TF fabrication, as well as users fitted by CP’s post training. The study group consisted of both users who needed a replacement prosthetic interface, according to new referral, due to wear and tear or volume changes (n=28; 26 analyzed; “clinical need”), and those with no clinical need of replacement (n=10) (Table 2). Study investigators indicate their criteria to determine if a new interface was clinically justified included: volume reduction requiring 5-7 sock ply or more (>7% volume reduction),^43^ socket discomfort, socket instability or significant skin irritation. The Mean age of subjects was 59 years (36-79 years) and represented wide range of activity levels. Study subjects exhibited baseline activity levels from K-level 1 to K-level 4, (K-level 1, n=4; K-level 2, n=11; K-level 3, n=21; and finally K-level 4, n=11).

**Table 2:** Characteristics of the study population stratified into follow-up periods according to those prosthetic users in need or not in need of a new interface.

	**Baseline**	**6WFU**	**6MFU**
**N**	47	40	38
**Men/Women**	33/14	29/11	28/10
Age (SD) in years	58.9 (11.8)	58.3 (11.7)	58.0 (12.0)
**Subjects in need of new interface**	34	29	28 (-2)^**^
Age (SD) in years)	59.0 (11.8)	58.2 (11.8)	58.1 (12.2)
Average K-level^*^	2.7 (0.9)	3.0 (0.8)	3.0 (0.9)
**Subjects not in need of new interface**	13	11	10
Age (SD) in years	58.8 (12.3)	58.5 (11.9)	57.9 (12.5)
Average K-level^*^	3.0 (0.8)	3.3 (01.0)	3.3 (0.8)

Age is referred to as means + SD

* K-level 1, K-level 2, K-level 3, K-level 4

** Two subject did not complete all measurement at all three time periods

Data was collected and analysed for 47 subjects at baseline. At 6WFU the results from 40 subjects were included and 6MFU included results from 38 subjects (Figure 3), providing a power of 95.3% and 95.2% for the follow-ups, respectively. Two subjects, from the group “with clinical need for new interface” did not complete the OPUS instrument at all three measurement time periods and are therefore not included in the data analysis for 6MFU.

All prosthetic users included in the study used a liner with their interface. Thirty-three of them used Seal-In liners, 5 used locking liners, 5 used lanyard and the rest used various types of liners and suspensions. The most frequent liner size was 35cm (range 25-50cm). While 36 of the users used microprocessor-controlled prosthetic knees from 2 different manufacturers, 11 knees were non-microprocessor-controlled prosthetic knees from 3 different manufacturers. Subjects’ prosthetic feet included extensive functional variation depending on user K-level, from a SACH foot to high activity feet, made by 4 different manufacturers. All subjects retained their existing knee and/or foot, except for two subjects that received a new knee and foot with the new interface.

Nine of the subjects dropped out of the study, 7 of them before 6WFU (including one deceased) and 2 of them before 6MFU (Figure 3). One subject who had advanced vascular disease and a very small limb decided to withdraw from the study after one week. Three subjects withdrew from study, preferring their previous socket.

One subject had shoulder surgery and was non-ambulatory for a significant part of the study for reasons not having to do with the prosthesis. Since the subject did not fulfil all steps of the study, he was considered a drop-out, however, the subject was still using the new interface when the study ended. Three subjects did not respond to follow-up. At least two of them continue to use the new DS-TF interface.

Each DS-TF interface was fabricated, fit and delivered in a single clinic visit. 36% of subjects surveyed at 6WFU had required minor adjustments by the study CP to the DS-TF between initial fitting and the 6WFU.

### Satisfaction assessment of all participants (Table 3 A,B)

**Table 3:** **A:** OPUS Client Satisfaction Device (CSD) (questions 1-11). **B:** OPUS Client Satisfaction with Services (CSS) (questions 12-21)

**A**				
	**Baseline**	**6WFU**	**6MFU**	
**All subjects**	**(n=47)**	**(n=41)**	**(n=36)**	***P***
1. My prosthesis fits well…	3.0 (1.2)^*^	4.3 (1.0)	4.6 (0.9)	<.001
2. The weight of my prosthesis is manageable…	3.8 (1.1)	4.5 (0.8)	4.5 (0.8)	<.001
3. My prosthesis is comfortable throughout the day…	3.0 (1.3)	4.0 (1.2)	4.4 (0.9)	<.001
4. It is easy to put on my prosthesis…	3.7 (1.1)	4.4 (0.9)	4.6 (0.6)	<.001
5. My prosthesis looks good…	3.8 (1.1)	4.2 (0.9)	4.7 (0.6)	<.001
6. My prosthesis is durable…	4.0 (1.1)	4.4 (0.9)	4.7 (0.5)	<.001
7. My clothes are free of wear and tear from my prosthesis…	3.3 (1.4)	4.1 (1.0)	4.1 (1.1)	<.001
8. My skin is free of abrasions and irritations…	3.1 (1.4)	4.0 (1.1)	4.0 (1.1)	<.001
9. My prosthesis is pain free to wear…	2.8 (1.3)	3.7 (1.0)	4.1 (1.2)	<.001
10. I can afford the out-of-pocket expenses to purchase and maintain my prosthesis	2.6 (1.4)	3.3 (1.5	2.7 (1.5)	0.91
11. I can afford to repair or replace my prosthesis as soon as needed	2.5 (1.5)	3.1 (1.6)	2.6 (1.5)	0.91
**B**				
12. I received an appointment with a prosthetist within a reasonable amount of time…	4.6 (0.7)	4.8 (0.4)	4.9 (0.4)	0.03
I was shown the proper level of courtesy and respect by the staff…	4.8 (0.4)	4.9 (0.5)	5.0 (0.2)	0.25
14. I waited a reasonable amount of time to be seen…	4.6 (0.9)	4.9 (0.4)	4.9 (0.3)	0.03
15. Clinic staff fully informed me about equipment choices…	4.6 (0.7)	4.9 (0.3)	4.9 (0.2)	0.03
16. The prosthetist gave me the opportunity to express my concerns regarding my equipment…	4.8 (0.5)	4.9 (0.3)	4.9 (0.3)	0.15
17. The prosthetist was responsive to my concerns and questions….	4.8 (0.4)	5.0 (0.2)	4.9 (0.3)	0.25
18. I am satisfied with the training I received in the use and maintenance of my prosthesis…	4.7 (0.5)	5.0 (0.2)	4.7 (0.9)	0.95
19. The prosthetist discussed problems I might encounter with my equipment…	4.7 (0.5)	5.0 (0.0)	4.9 (0.4)	0.13
20. The staff coordinated their services with my therapists and doctors…	4.5 (0.8)	4.4 (1.3)	4.9 (0.3)	0.15
21. I was a partner in decision-making with clinic staff regarding my care and equipment…	4.7 (0.5)	4.9 (0.3)	4.9 (0.3)	0.08

* All data are presented as mean (SD)

Average CSD scores (the sum of questions 1-11 on a 100-point Rasch Measure scale) for participants that completed the 6MFU (n=36) were:

- 46.9 (SD=9.8) at baseline (i.e. existing interface/prosthesis)
- 60.6 (SD=14.5) at 6WFU, an increase of 29.8 (SD=3.3)% compared to baseline with P<.001
- 61.0 (SD=14.0) at 6MFU, an increase of 29.8 (SD=3.3)%, compared to baseline, with P<.001

Each question directly related to the use of the new interface (items 1-9), showed significant improvement in outcome, with the main difference between baseline and 6WFU. Improvement was sustained in all 9 questions between 6WFU and 6MFU (Table 3 A for individual scores; F statistic = 29.1 degrees of freedom = 5). No study subject responded to any survey question with the selection "Don't Know / Not Applicable".

Mixed effect model analysis showed that “clinical need” did not affect the CSD measure (Chi squared = 0.27, P = 0.60).

Average CSS scores (the sum of questions 12-21 on a 100 point Rasch Measure scale) for participants that completed the 6MFU (n=36) were:

- 81.3 (SD=19.9) at baseline
- 90.6 (Sd=14.1) at 6WFU, an increase of 12.3 (SD=2.2)% compared to baseline with P=0.009
- 93.1 (SD=13.8) at 6MFU, an increase of 14.8 (SD=2.2)% compared to baseline with P=0.001

See Table 3B for individual scores; F statistic = 8.2; degrees of freedom = 5). Mixed effect model analysis showed that “clinical need” did not affect the CSS measure (Chi squared = 0.04, P=0.85).

### Satisfaction assessment of participants with the clinical need for new interface (Table 4 A,B)

**Table 4:** **A:** OPUS Client Satisfaction Device (CSD) (questions 1-11); **B:** OPUS Client Satisfaction with Services (CSS) (questions 12-21).

**A**	**Baseline**	**6WFU**	**6MFU**
**Subject with clinical need**	**(n=26)**	**(n=26)**	**(n=26)**
1. My prosthesis fits well…	2.8 (1.2)	4.3 (0.8)	4.6 (1.0)
2. The weight of my prosthesis is manageable…	3.6 (1.2)	4.4 (0.9)	4.5 (0.9)
3. My prosthesis is comfortable throughout the day…	2.8 (1.4)	4.0 (1.1)	4.3 (1.0)
4. It is easy to put on my prosthesis…	3.6 (1.2)	4.4 (0.9)	4.7 (0.6)
5. My prosthesis looks good…	3.7 (1.1)	4.2 (1.1)	4.7 (0.5)
6. My prosthesis is durable…	3.8 (1.4)	4.3 (1.0)	4.7 (0.5)
7. My clothes are free of wear and tear from my prosthesis…	3.2 (1.4)	4.2 (1.0)	4.3 (0.9)
8. My skin is free of abrasions and irritations…	3.3 (1.4)	4.2 (0.8)	4.2 (1.0)
9. My prosthesis is pain free to wear…	2.6 (1.2)	3.8 (1.0)	4.2 (1.1)
10. I can afford the out-of-pocket expenses to purchase and maintain my prosthesis…	2.5 (1.4)	3.5 (1.5)	2.7 (1.5)
11. I can afford to repair or replace my prosthesis as soon as needed	2.5 (1.5)	3.3 (1.6)	2.5 (1.4)
**B**			
12. I received an appointment with a prosthetist within a reasonable amount of time…	4.7 (0.5)	4.8 (0.4)	4.9 (0.4)
13. I was shown the proper level of courtesy and respect by the staff…	4.8 (0.4)	4.8 (0.6)	5.0 (0.2)
14. I waited a reasonable amount of time to be seen…	4.6 (0.9)	4.8 (0.5)	4.9 (0.3)
15. Clinic staff fully informed me about equipment choices…	4.6 (0.8)	4.9 (0.3)	4.9 (0.3)
16. The prosthetist gave me the opportunity to express my concerns regarding my equipment…	4.7 (0.5)	4.8 (0.4)	4.9 (0.3)
17. The prosthetist was responsive to my concerns and questions….	4.8 (0.4)	5.0 (0.2)	4.9 (0.3)
18. I am satisfied with the training I received in the use and maintenance of my prosthesis…	4.7 (0.5)	5.0 (0.2)	4.9 (0.3)
19. The prosthetist discussed problems I might encounter with my equipment…	4.7 (0.5)	5.0 (0.0)	4.8 (0.5)
20. The staff coordinated their services with my therapists and doctors…	4.5 (0.9)	4.5 (1.4)	4.9 (0.3)
21. I was a partner in decision-making with clinic staff regarding my care and equipment…	4.6 (0.5)	4.9 (0.3)	4.9 (0.3)

For participants that completed the 6MFU (n=26)

*Average CSD scores were:*

- 45.5 (SD=9.1) at baseline
- 60.5 (SD=14.0) at 6WFU, an increase of 33.3 (SD=3.9) % with P<.001
- 61.9 (SD=14.1) at 6MFU, an increase of 37.8 (SD=4.0) %, compared to baseline with P<.001

*Average CSS scores were:*

- 80.8 (SD=20.2) at baseline
- 91.0 (SD=15.2) at 6WFU, an increase of 12.3 (SD=3.4) % with P=0.055
- 93.3 (SD=13.4) at 6MFU, an increase of 14.8 (SD=3.4)% compared to baseline with P<.005.

### Satisfaction assessment of participants without the clinical need for new interface (Table 5 A,B)

**Table 5:** **A:** OPUS Client Satisfaction Device (CSD) (questions 1-11); **B:** OPUS Client Satisfaction with Services (CSS) (questions 12-21).

**A**	**Baseline**	**6WFU**	**6MFU**
**Subject with no clinical need**	**(n=10)**	**(n=10)**	**(n=10)**
1. My prosthesis fits well…	3.6 (0.8)	4.7 (0.7)	4.5 (0.5)
2. The weight of my prosthesis is manageable…	4.5 (0.5)	4.9 (0.3)	4.6 (0.5)
3. My prosthesis is comfortable throughout the day…	3.5 (1.0)	4.5 (0.7)	4.4 (0.7)
4. It is easy to put on my prosthesis…	3.9 (0.7)	4.8 (0.4)	4.6 (0.7)
5. My prosthesis looks good…	4.0 (0.7)	4.5 (0.7)	4.6 (0.7)
6. My prosthesis is durable…	4.3 (0.7)	4.7 (0.7)	4.6 (0.7)
7. My clothes are free of wear and tear from my prosthesis…	3.5 (1.4)	4.3 (1.1)	3.7 (1.3)
8. My skin is free of abrasions and irritations…	3.0 (1.2)	3.6 (1.4)	3.6 (1.2)
9. My prosthesis is pain free to wear…	3.4 (1.3)	3.6 (1.1)	3.7 (1.3)
10. I can afford the out-of-pocket expenses to purchase and maintain my prosthesis…	2.7 (1.4)	2.6 (1.5)	2.8 (1.6)
11. I can afford to repair or replace my prosthesis as soon as needed	2.4 (1.4)	2.6 (1.6)	2.9 (1.7)
**B**			
12. I received an appointment with a prosthetist within a reasonable amount of time…	4.4 (1.0)	4.7 (0.5)	5.0 (0.0)
13. I was shown the proper level of courtesy and respect by the staff…	4.8 (0.4)	5.0 (0.0)	5.0 (0.0)
14. I waited a reasonable amount of time to be seen…	4.5 (1.0)	5.0 (0.0)	5.0 (0.0)
15. Clinic staff fully informed me about equipment choices…	4.8 (0.4)	4.9 (0.3)	5.0 (0.0)
16. The prosthetist gave me the opportunity to express my concerns regarding my equipment…	4.8 (0.4)	5.0 (0.0)	5.0 (0.0)
17. The prosthetist was responsive to my concerns and questions….	4.8 (0.4)	5.0 (0.0)	4.9 (0.3)
18. I am satisfied with the training I received in the use and maintenance of my prosthesis…	4.7 (0.5)	5.0 (0.0)	4.3 (1.7)
19. The prosthetist discussed problems I might encounter with my equipment…	4.7 (0.5)	5.0 (0.0)	5.0 (0.0)
20. The staff coordinated their services with my therapists and doctors…	4.6 (0.5)	4.4 (1.1)	5.0 (0.0)
21. I was a partner in decision-making with clinic staff regarding my care and equipment…	4.8 (0.4)	4.9 (0.3)	4.9 (0.3)

For participants that completed the 6MFU (n=10)

*Average CSD scores were:*

- 50.7 (SD=11.1) at baseline
- 61 (SD=16.4) at 6WFU, an increase of 19.6 (SD=4.5)% with P=0.018
- 58.7 (SD=14.0) at 6MFU, an increase of 15.6 (SD=4.9) % compared to baseline with P=0.047

*Average CSS scores were:*

- 82.8 (SD=20.0) at baseline
- 89.5 (SD=11.6) at 6WFU, an increase of 8.8 (SD=7.5)% compared to baseline with P=0.195
- 92.6 (SD=15.5), at 6MFU, an increase of 12.0 (SD=7.9)% compared to baseline with P= 0.103.

## DISCUSSION

OPUS CSD questions related to the function of the interface for all subjects indicate a significant improvement in user satisfaction with their DS-TF interface over their previous interface in terms of weight, comfort, donning, appearance, durability, and reduced clothing wear and tear. Results also showed significantly improved satisfaction regarding skin abrasions and irritation, as well “pain free to wear”. All improvements were consistent between the 6-week and 6-month study periods. At 6MFU the average CSS score was 93, or 14.8% higher, a significant improvement compared with baseline.

One might think that amputees who need a new socket for clinical reasons may be more dissatisfied with their existing socket than amputees who do not need a new socket for clinical reasons, and might therefore be more inclined to show greater satisfaction improvement with a new prosthesis than the subjects who did not need a new interface. To identify this potential influence, all completed subject data was analyzed together, as well as broken into two separate cohorts- “with clinical need of new interface” and “without clinical need of new interface”. Analysis indicates that “clinical need” did not significantly affect the CSD and CSS measures.

Across both user groups average CSD OPUS scores were significantly improved after fitting subjects with DS-TF interfaces. In all cases, the CP was able to fabricate, align, fit, adjust, and deliver the new interface in the same visit, with only 36% of subjects returning to the clinic for a post-delivery socket adjustment.

To our knowledge this study is one of largest and longest prosthetic user satisfaction outcome studies published to date with focus on a new interface design. In this study we followed 38 TF amputees, comprised of household ambulators up to high active users (K1 to K4) for 6-months. Our observed 19% drop-out rate (9 out of 47) can be expected in such a group over multiple data collection intervals and 6-months’ time.^40^

We believe the improved user satisfaction with service is from two factors that DS-TF enables: 1) single-visit fabrication and delivery, and 2) that subjects feel more “involved” in their socket fabrication. While it is possible that baseline OPUS scores may include subject recall bias since patients received their existing prosthesis and interfaces months or years in the past, we believe a comparison is informative.

As the DS-TF fabrication and delivery can usually be completed in a single (but longer) clinic visit, DS-TF eliminates or reduces the hassle of multiple trips to the clinic by the amputee and family or care givers. During the current Covid-19 pandemic, TF amputees may especially value fewer clinic visits in order to reduce their risk of infection. CP’s fabricate DS-TF sockets directly on the residual limb; anecdotal subject reports indicate users enjoyed the opportunity to be more involved in the entire process, to see each step, to communicate with their CP and be a part of design decisions (e.g. the position of the brim, how to manage sensitive areas, placement of the valve, etc.).

In liquid form the two-part resin used in DS-TF fabrication can cause injury to amputee and clinicians if handled improperly. It is therefore critical to follow safe DS-TF fabrication procedures using protective equipment for clinicians and amputee which means process training and practice of DS-TF fabrication should never be underrated.^34^

The current standard of care for TF interface design focuses on the proximal aspect of the socket to achieve a stable stance-phase connection between socket and amputee. The proximal socket brim extends above the femur and is intended to contain the ischial ramus, thereby preventing a lateral shift of the socket and enhancing user stability.^26^ To deliver a finished laminated TF interface of this type most CP's use a complicated multi-step fabrication process including: hand casting, one or more test sockets, one or more laminated sockets, and usually at least one post-fitting adjustment where patient comes back to the clinic. However, this socket design has never been described using the ISO standard 13405-2:2015. This method is also dependent on many years of CP experience and rarely based on evidence and/or outcome studies.

The direct casting method used in this study is a different concept and process, unlike most CP’s traditional processes. The DS-TF socket is a true transfemoral socket as no rigid part of the DS-TF socket bears load from the ischium bone. DS-TF instead focuses on a unique way of supporting the hip joint through the hip muscles; i.e. when the hip muscles contract and expand during stance phase, the DS-TF brim supports the hip muscles. The support that the DS-TF brim provides, activates and stimulates important hip muscle function^44^ to enable axial and transverse stability during normal walking.^23^ These different interfaces (their function and design) should follow standardized method when they are described, this to enhance baseline comparison.^23^

It should be noted that the cost of prosthetic provision has been shown to be less than 10% of the total cost of an amputation,^45^ with delayed rehabilitation adding considerable expense to the total cost.^2^ It has also been demonstrated that less time between amputation and weight bearing or ambulation therapy can both contribute to faster restoration of the walking ability after a lower limb amputation^46^ and reduce the cost of rehabilitation of these patients.^2^ Using DS-TF a prosthetist can potentially reduce the time between amputation and start of weight bearing and physical therapy down to 6 weeks as shown in study when using the DS-TT.^13^ DS-TF can be an important tool to improve lower limb amputee outcomes and potentially reducing the overall health care costs to society of treating transfemoral amputees.

### Limitations

Study subjects received their existing interfaces months or years in the past. Therefore, having users complete the OPUS on their existing socket introduces the possibility of recall bias. Primary study limitation is not having the amputees randomized into DS-TF (intervention) and control (traditional interface) groups. Dividing the cohort into groups based on “clinical need” for a new interface versus no clinical need for a new interface was a partial mitigation of this limitation. However, the aim of this study was to evaluate the implementation of direct fabrication on the amputee’s limb and to gather subject outcomes over an extended and clinically meaningful time.

New amputees often take an extended time to adjust to the interface, therefore we suggest that future studies would follow new users over one year, randomized into two groups of traditional interfaces versus DS-TF interfaces. Several study subjects provided anecdotal reports of significantly reduced phantom pain, therefore another potential area of further research would be to investigate the impact of DS-TF on phantom pain.

## CONCLUSION

This study shows that following a standardized training and implementation plan, the DS-TF process can be successfully applied in caring for TF prosthetic users. The unique DS-TF interface design yields greater user satisfaction regarding interface function and comfort compared to a traditional TF interface design. The novel DS-TF interface fabrication method also yields increased user satisfaction with the CPs’ fabrication and delivery of the interface and prosthesis compared to the service users received along with their previous interface.

## DECLARATION OF CONFLICTING INTERESTS

All authors are employees of Össur HF except I. F. Atlason. Study Principle Investigators received no compensation from Össur HF.

## AUTHOR CONTRIBUTION

- **W. Russ Marable:** Conceptualization; Study oversight; Data collection; Writing original; Review and editing
- **Christian Smith:** Conceptualization; Data collection; Writing original; Review and editing
- **Benedikt Þorri Sigurjonsson:** Conceptualization; Obtained funding; Study oversight; Data analysis; Review and editing
- **Ingi Freyr Atlason:** Data analysis
- **G. Anton Johannesson:** Conceptualization; Study oversight; Data analysis; Writing original; Review and editing

## SOURCES OF SUPPORT

This study was financially supported by Össur HF.

## ETHICAL APPROVAL

Ethical approval was obtained from Advarra^®^ IRB (CR00128417) and the investigation was registered at Clinical trials.gov NCT04312724. Signed informed consent was obtained from all participants.
